# Supplementary material for: Histone methyltransferase PRDM9 promotes survival of drug-tolerant persister cells in glioblastoma
Source: Nat Commun. 2025 Dec 15;16:10905. doi: 10.1038/s41467-025-65888-5 (PMC12705669; doi:10.1038/s41467-025-65888-5)
Supplement: Supplementary file 1 — Supplementary Information [file 41467_2025_65888_MOESM1_ESM.pdf]

Histone methyltransferase PRDM9 promotes survival of drug-tolerant persister cells in glioblastoma

George L. Joun[1,2], Emma G. Kempe[1,2], Brianna Chen[1,2], Jayden R. Sterling[1,2], Ramzi H. Abbassi[1,2], Dana Friess[3,4], Matthew Singleton[3], Chandra Choudhury[3,4], Oana C. Marian[1,2], W. Daniel du Preez[1,2], Ariadna Recasens[1,2], Teleri Clark[2,5], Tian Y. Du[2,5], Jason K.K. Low[5], Hani Kim[2,6], Pengyi Yang[2,6], Jasmine Khor[1,2], Monira Hoque[1,2], Dinesh C. Indurthi[1,2], Mani Kuchibhotla[7,8], Ranjith Palanisamy[7,8], William T. Jorgensen[9], Andrew P. Montgomery[9], Jennifer R. Baker[10], Sarah L. Higginbottom[11,12], Eva Tomaskovic-Crook[1,11,12], Jeremy M. Crook[1,11,12], Lipin Loo[2,5], Bryan W. Day[3], G. Gregory Neely[2,5], Ernesto Guccione[13,14,15], Terrance G. Johns[7,8], Michael Kassiou[9], Yuchen Feng[1,2], Lachlan Harris[3,4,16], Anthony S. Don[1,2], Lenka Munoz[1,2*]

SUPPLEMENTARY INFORMATION

SUPPLEMENTARY FIGURE 1-9: pages 2 - 10
SUPPLEMENTARY TABLE 1-3: pages 11 - 12

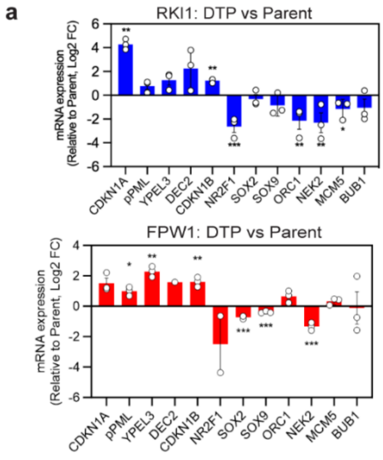

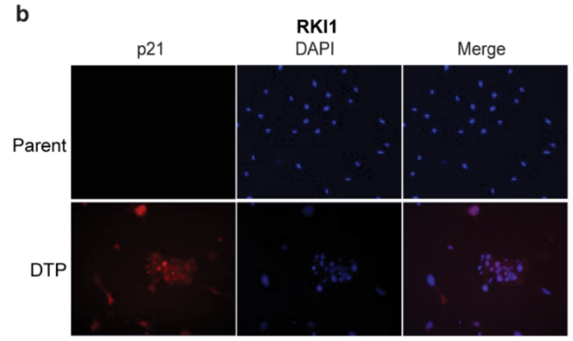

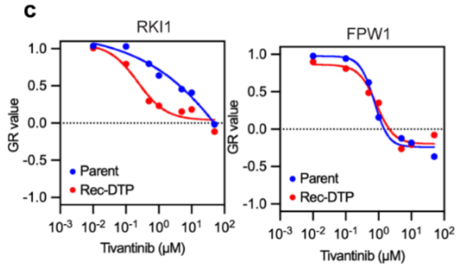

d

Parent RKI1 cells

Drug	CMPD1	Tivantinib
GR_{50}	1.97 ± 0.510	3.44 ± 0.325
GR_{max}	-0.01 ± 0.031	-0.02 ± 0.089
h_{GR}	1.06 ± 0.224	0.64 ± 0.083
GR_{AOC}	0.37 ± 0.065	0.31 ± 0.029

Parent FPW1 cells

Drug	CMPD1	Tivantinib
GR_{50}	0.70 ± 0.114	0.59 ± 0.037
GR_{max}	-0.29 ± 0.039	-0.37 ± 0.002
h_{GR}	2.26 ± 0.241	1.74 ± 0.455
GR_{AOC}	0.61 ± 0.049	0.62 ± 0.012

Recovered RKI1 DTPs

Drug	CMPD1	Tivantinib
GR_{50}	1.76 ± 0.395	0.29 ± 0.057
GR_{max}	0.07 ± 0.033	-0.11 ± 0.034
h_{GR}	0.39 ± 0.019	0.57 ± 0.026
GR_{AOC}	0.82 ± 0.076	1.32 ± 0.089

Recovered FPW1 DTPs

Drug	CMPD1	Tivantinib
GR_{50}	1.10 ± 0.607	1.09 ± 0.645
GR_{max}	-0.38 ± 0.096	-0.18 ± 0.071
h_{GR}	1.55 ± 0.666	0.65 ± 0.165
GR_{AOC}	0.49 ± 0.038	0.52 ± 0.088

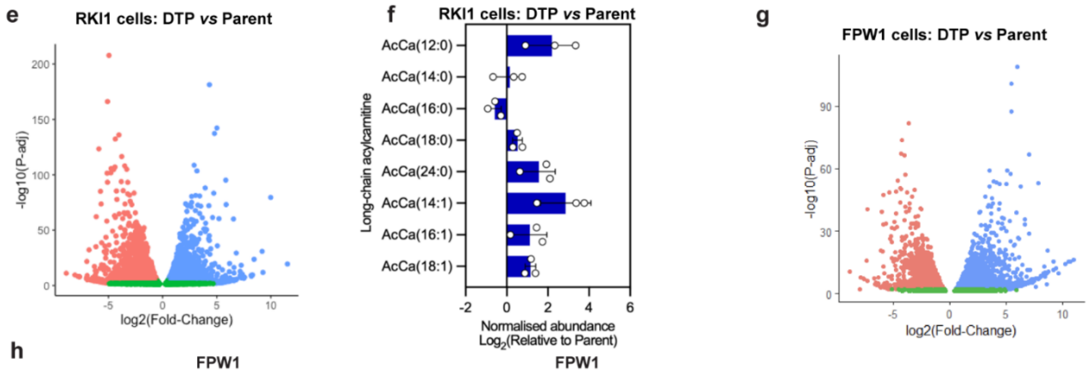

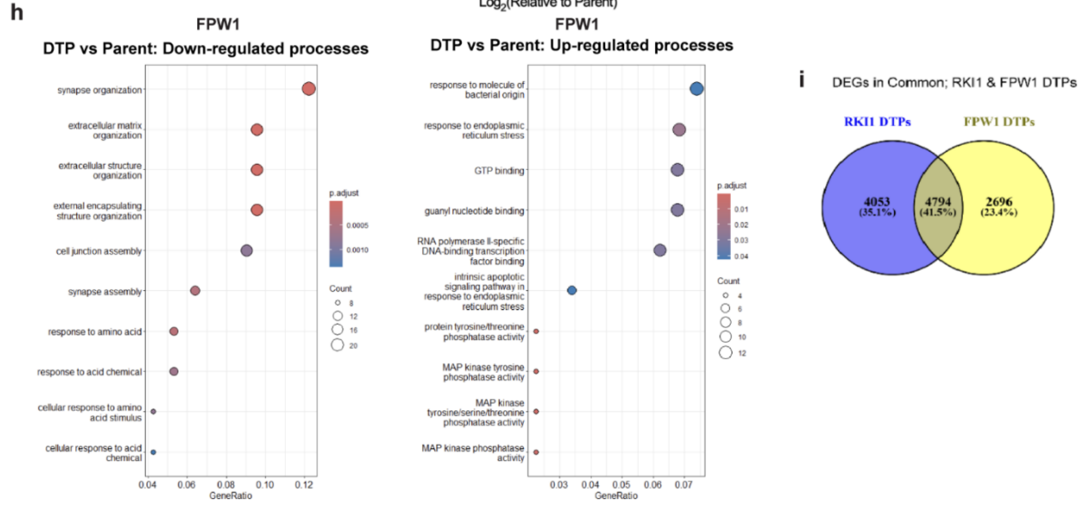

Supplementary Figure 1. **a** mRNAs in CMPD1 (25 μM, 14 days) derived drug-tolerant persister (DTP) cells compared to parent cells. Data are mean ± SD (n = 3 biological replicates). One sample t-test: * indicates p (two tailed) < 0.05; ** indicate p (two tailed) < 0.01, *** indicates p (two tailed) < 0.001. **b** Imaging of p21 in parent and CMPD1 (25 μM, 14 days) derived DTP cells. **c** Tivantinib dose response curves in parent and recovered DTP cells (generated with tivantinib (25 μM, 14 days), followed by drug holiday). Data are mean of n = 3 biological replicates. **d** GR metrics for CMPD1 and tivantinib in parent and recovered DTP cells, values were calculated from curves in Figure 1c and Supplementary Figure 1c. **e** Volcano plot of differentially expressed genes (DEGs, via RNA-seq of n = 3 biological replicates) in CMPD1 (25 μM, 14 days) derived DTP cells compared to parent RKI1 cells. Down-regulated DEGs are in red, upregulated DEGs in blue, non-significant genes in green. **f** Fold-change of long-chain carnitines in CMPD1 (25 μM, 14 days) derived DTP cells compared to parent cells. Data are mean ± SD (n = 3). **g** Volcano plot of DEGs (via RNA-seq of n = 3 biological replicates) in CMPD1 (25 μM, 14 days) derived DTP cells compared to parent FPW1 cells. Down-regulated DEGs are in red, upregulated DEGs in blue, non-significant genes in green. **h** Gene Ontology of top 200 down- and upregulated genes in CMPD1 (25 μM, 14 days) derived DTP cells compared to FPW1 parent cells. **i** Venn diagram of DEGs in CMPD1 (25 μM, 14 days) derived DTP cells compared to parent cells. Source data are provided as a Source Data file.

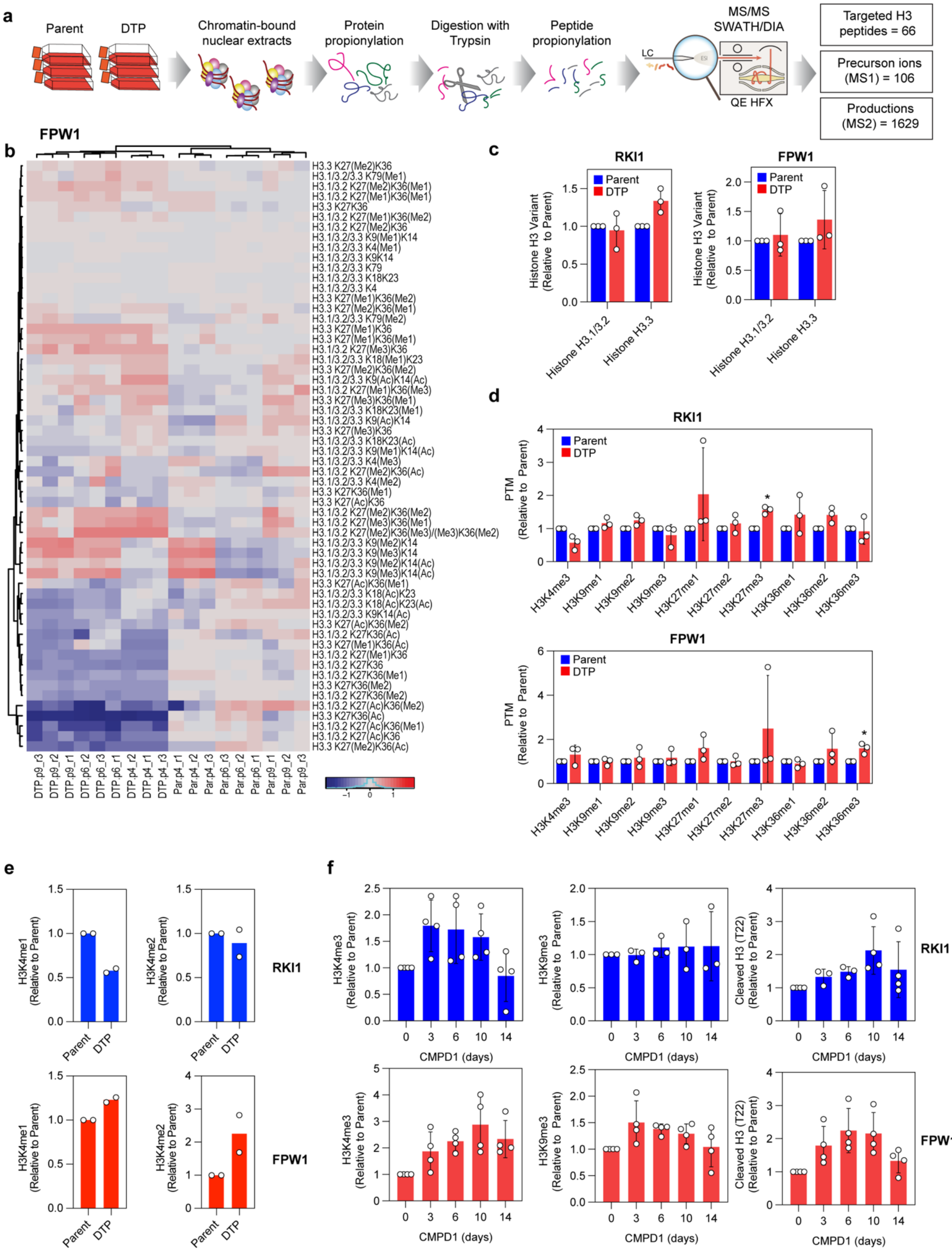

Supplementary Figure 2. **a** Schematic of mass spectrometry workflow for histone proteomics in Figure 2. **b** Heatmap of peak areas for 66 unmodified, methylated and acetylated H3 peptides in FPW1 parent and CMPD1 (25 µM, 14 days) derived drug-tolerant persister (DTP) cells. Data are parent-DTP pairs of 3 biological replicates; each performed in triplicate. **c** Quantification of H3.1/H3.2 and H3.3 variants normalised to total H3 in parent and CMPD1 (25 µM, 14 days) derived DTP cells (related to Figure 2c). Data are mean ± SD (n = 3 biological replicates). **d** Quantification of histone post-translational modifications (PTM) normalised to total H3 in parent and CMPD1 (25 µM, 14 days) derived DTP cells (related to Figure 2f). Data are mean ± SD (n = 3 biological replicates). One sample t-test: * indicates p (two tailed) = 0.0125, p (two tailed) = 0.0497 for H3K27me3 and H3K36me3, respectively. **e** Quantification of H3K4me1 and H3K4me2 normalised to total H3 in parent and CMPD1 (25 µM, 14 days) derived DTP cells (related to Figure 2g). Data are mean from n = 2 biological replicates. **f** Quantification of H3K4me3, H3K9me3 and cleaved H3 normalised to total H3 in CMPD1 (25 µM) treated RKI1 and FPW1 cells (related to Figure 2h). Data are mean ± SD (n = 3 biological replicates for H3K9me3 in RKI1 cells, n = 4 for all other panels). Source data are provided as a Source Data file.

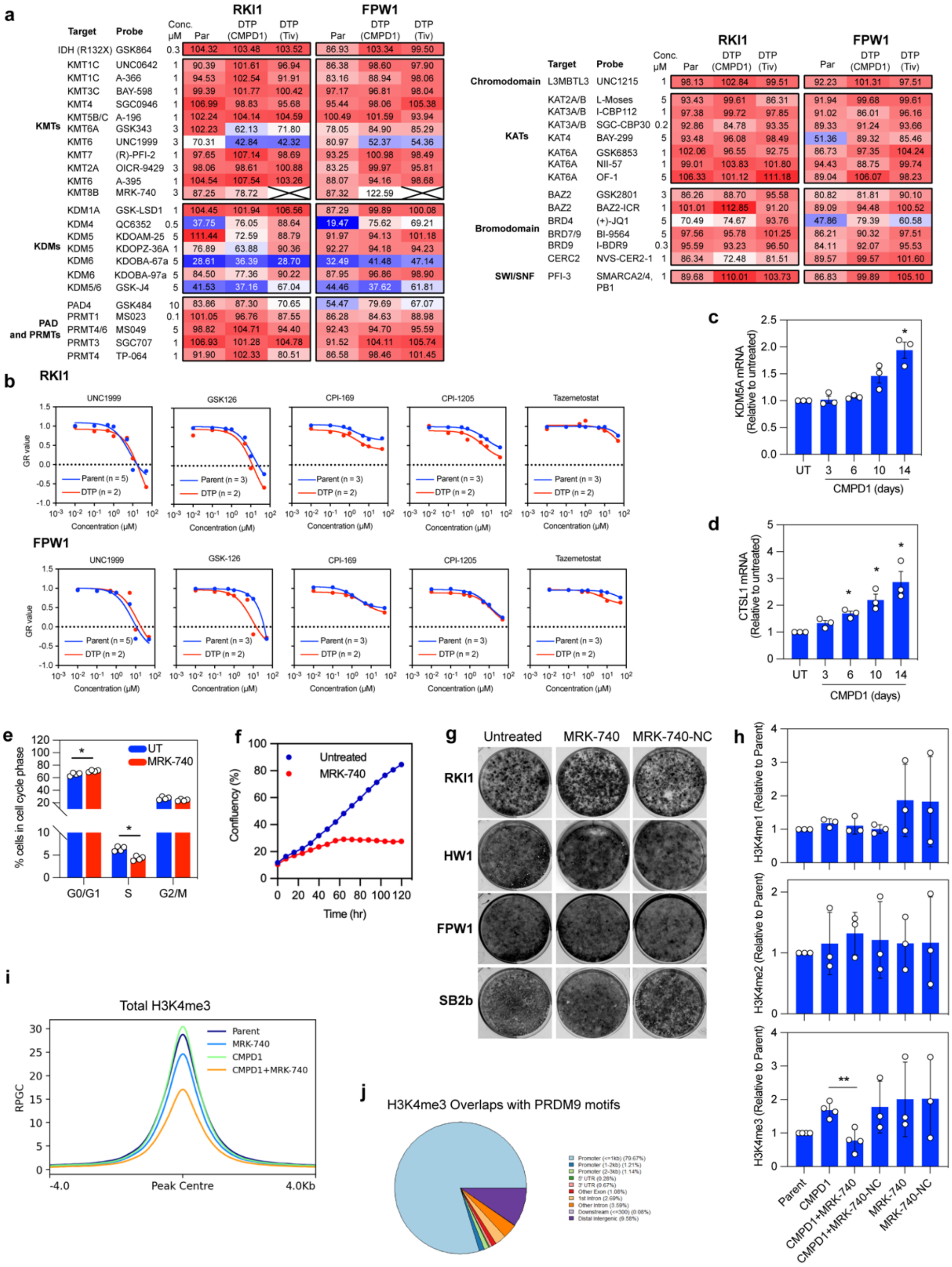

Supplementary Figure 3. a Drug-tolerant persisters (DTP; CMPD1 or tivantinib, 25 µM, 14 days) and Parent (Par) cells were treated with epigenetic probes, heatmap displays mean values (%) of cell viability at Day 5 relative to untreated cells. **b** DTP cells (CMPD1, 25 µM, 14 days) and Parent cells were treated with KMT6 inhibitors for 5 days, growth-rate (GR) values calculated using the *GRcalculator* tool. Data are mean of n biological replicates indicated in the figure. **c** KDM5A mRNA in RKI1 cells treated with CMPD1 (25 µM). Data are mean ± SD (n = 3 biological replicates). One sample t-test: * indicates p (two tailed) = 0.0249. **d** CTSL1 mRNA in RKI1 cells treated with CMPD1 (25 µM). Data are mean ± SD (n = 3 biological replicates). One sample t-test: * indicates p (two tailed) = 0.0168, p (two tailed) = 0.0321, p (two tailed) = 0.0437 for 6, 10,14 days, respectively. **e** Cell cycle distribution of RKI1 cells treated with MRK-740 (3 µM, 6 days). Data are mean ± SD (n = 4 biological replicates). Unpaired t-test: * indicates p (two tailed) = 0.0097, p (two tailed) = 0.0011 for G0/G1 and S phase, respectively. **f** Incucyte Live-Cell imaging of RKI1 cells treated with MRK-740 (3 µM). Each datapoint is the average of confluency of nine images per well. **g** Representative images (n = 3 biological replicates) of glioblastoma stem cell lines treated with MRK-740 (3 µM) or MRK-740-NC (3 µM, 7 days), followed by drug holiday, accompanying data in Figure 3g. **h** Quantification of H3K4me1, H3K4me2 and H3K4me3 in RKI1 cells treated with CMPD1 (10 µM, 3 days) ± MRK-740 (3 µM) ± MRK-740-NC (3 µM) (related to Figure 3i). Data are mean ± SD (n = 3 biological replicates). Unpaired t-test: ** indicates p (two tailed) = 0.004. **i** Total H3K4me3 ChIPseq signal intensity (RPGC) in RKI1 cells treated with CMPD1 (10 µM, 3 days) ± MRK-740 (3 µM). **j** Region annotations of H3K4me3 peaks overlapping with a PRDM9 motif, related to Figure 3j. Source data are provided as a Source Data file.

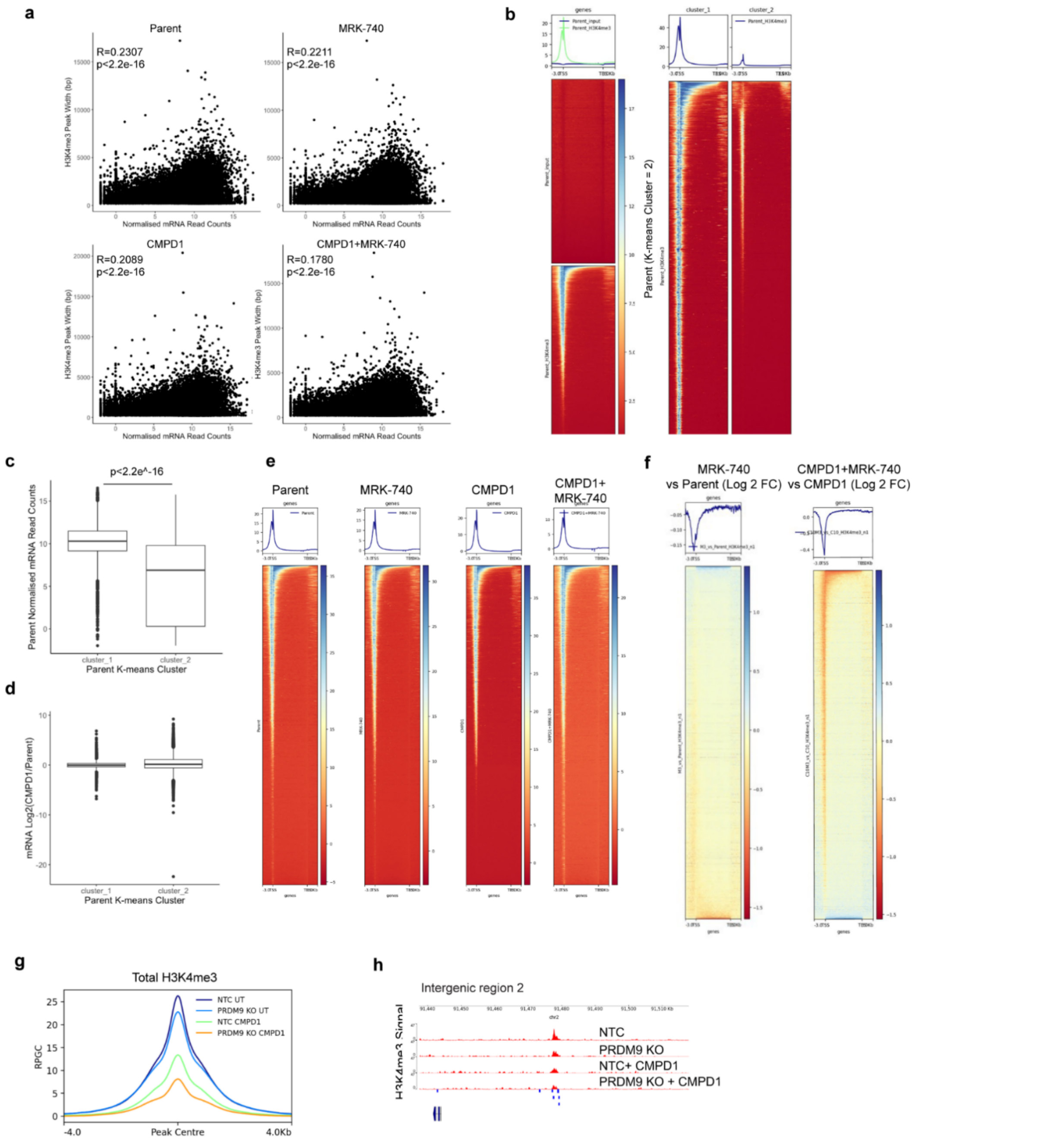

Supplementary Figure 4. a Scatter plots showing correlation between H3K4me3 peak width and normalised transcript counts (rlog). Pearson's product moment correlation coefficient was used to determine R value and statistical significance (P value). Parent: n = 21,521 H3K4me3 peaks; MRK-740: n = 21,305 H3K4me3 peaks; CMPD1: n = 21,794 H3K4me3 peaks; CMPD1+MRK-740: n = 22,472 H3K4me3 peaks; matched with corresponding transcripts. **b** Genome wide H3K4me3 enrichment heatmaps for parent RKI1 cells divided into two K-means clusters. Genome-wide H3K4me3 enrichment was profiled across n = 186,794 functional genomic regions (hg38). **c** mRNA changes in parent cells for H3K4me3 Cluster 1 vs Cluster 2 genes. mRNA transcripts were mapped to n = 54,933 genomic regions in Cluster 1 and n = 128,665 in Cluster 2 (from **b**); yielding n = 23,006 transcripts successfully mapped in Cluster 1 and n = 36,168 transcripts successfully mapped in Cluster 2. mRNA transcripts from RNA-seq experiment in Figure 5a. Box plots display the median as the centre line, the box representing the 25th - 75th percentiles, and the whiskers extending to 1.5x the interquartile range (IQR) beyond the box limits. Outliers are shown as individual data points. Wilcoxon rank-sum test was used to calculate P value. **d** mRNA changes (Log2 FC) in CMPD1 (10 µM, 3 days) treated vs parent RKI1 cells for H3K4me3 Cluster 1 and Cluster 2 genes. mRNA transcripts were mapped to n = 54,933 genomic regions in Cluster 1 and n = 128,665 in Cluster 2 (from **b**); yielding n = 23,006 transcripts successfully mapped in Cluster 1 and n = 36,168 transcripts successfully mapped in Cluster 2. mRNA transcripts derived from RNA-seq experiment in Figure 5a. Box plots display the median as the centre line, the box representing the 25th - 75th percentiles, and the whiskers extending to 1.5x the interquartile range (IQR) beyond the box limits. Outliers are shown as individual data points. Wilcoxon rank-sum test was used to calculate P value. **e, f** Genome wide heatmaps for H3K4me3 signal (± 3kb from transcription start/end site) in RKI1 cells treated with CMPD1 (10 µM, 3 days) ± MRK-740 (3µM). Genome-wide H3K4me3 enrichment was profiled across n = 186,794 functional genomic regions (hg38). **g** Total H3K4me3 ChIPseq signal density (n = 23,373 peaks; RPGC normalisation) in RKI1 cells transduced with NTC sgRNA (NTC) or PRDM9 sgRNA (PRDM9 KO) and treated with CMPD1 (10 µM, 3 days). **h** Individual genome track of H3K4me3 intensity (RPGC) at an intergenic region aligning with PRDM9 motif (blue line) in CMPD1 (10 µM, 3 days) treated RKI1 cells transduced with NTC sgRNA (NTC) or PRDM9 sgRNA (PRDM9 KO).

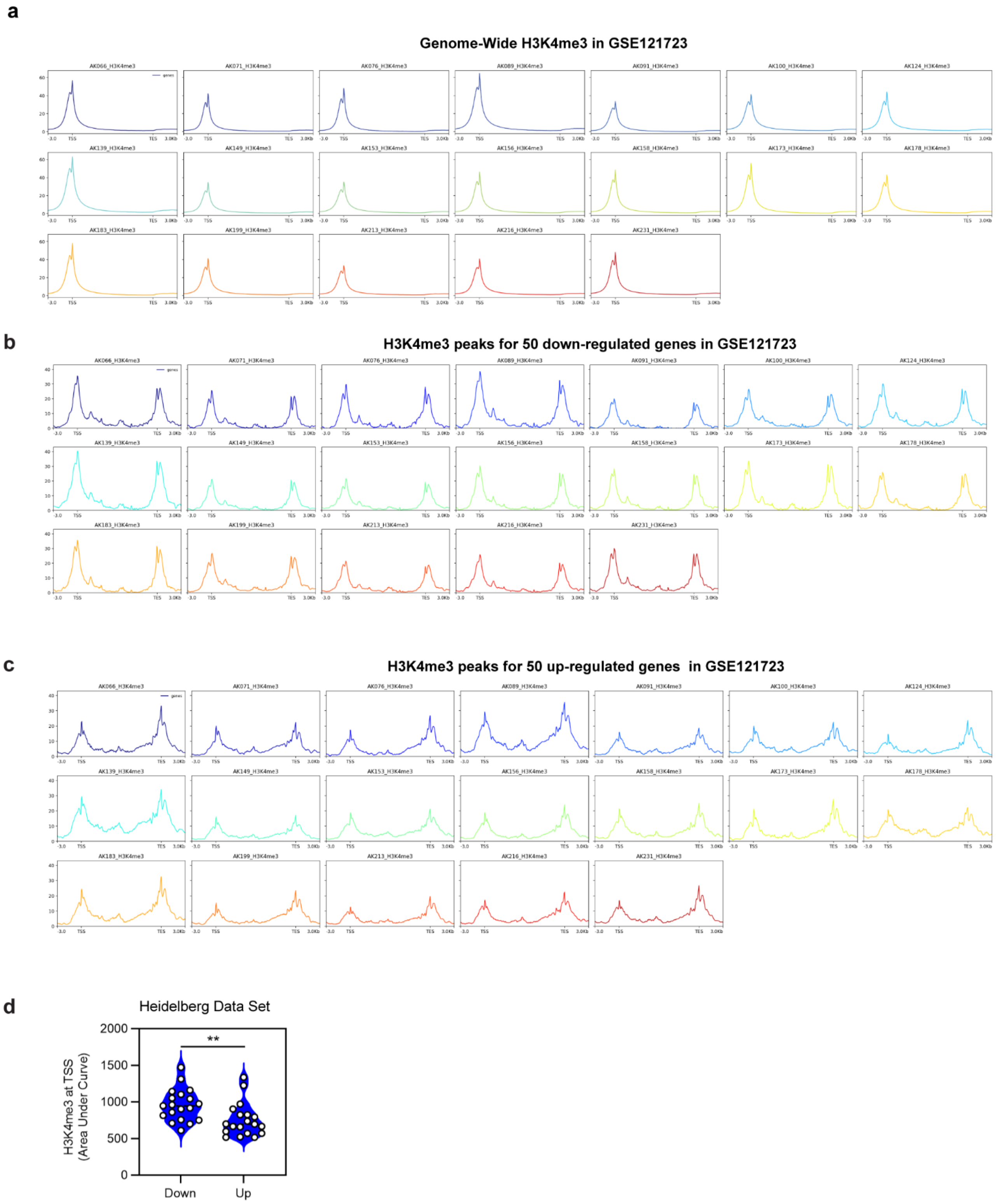

Supplementary Figure 5. a Genome wide H3K4me3 intensity (RPKM; ± 3kb from transcription start/end site) in 19 glioblastoma specimens (GSE121723)[1]. **b** H3K4me3 signal intensity (RPKM; ± 3kb from transcription start/end site) for down-regulated genes (listed in Figure 4a) in in 19 glioblastoma specimens (GSE121723)[1]. **c** H3K4me3 signal intensity (RPKM; ± 3kb from transcription start/end site) for up-regulated genes in 19 glioblastoma specimens (GSE121723)[1]. **d** Quantification of H3K4me3 peaks at the transcription start sites (TSS, ± 3kb from transcription start site) of down-regulated and up-regulated genes (identified in RKI1 cells: CMPD1+MRK-740 vs CMPD1) in 19 glioblastoma specimens (GSE121723)[1]. The width of the violin represents the kernel density estimate, illustrating the distribution of the data. Unpaired t-test: ** indicates p (two tailed) = 0.0077. Source data are provided as a Source Data file.

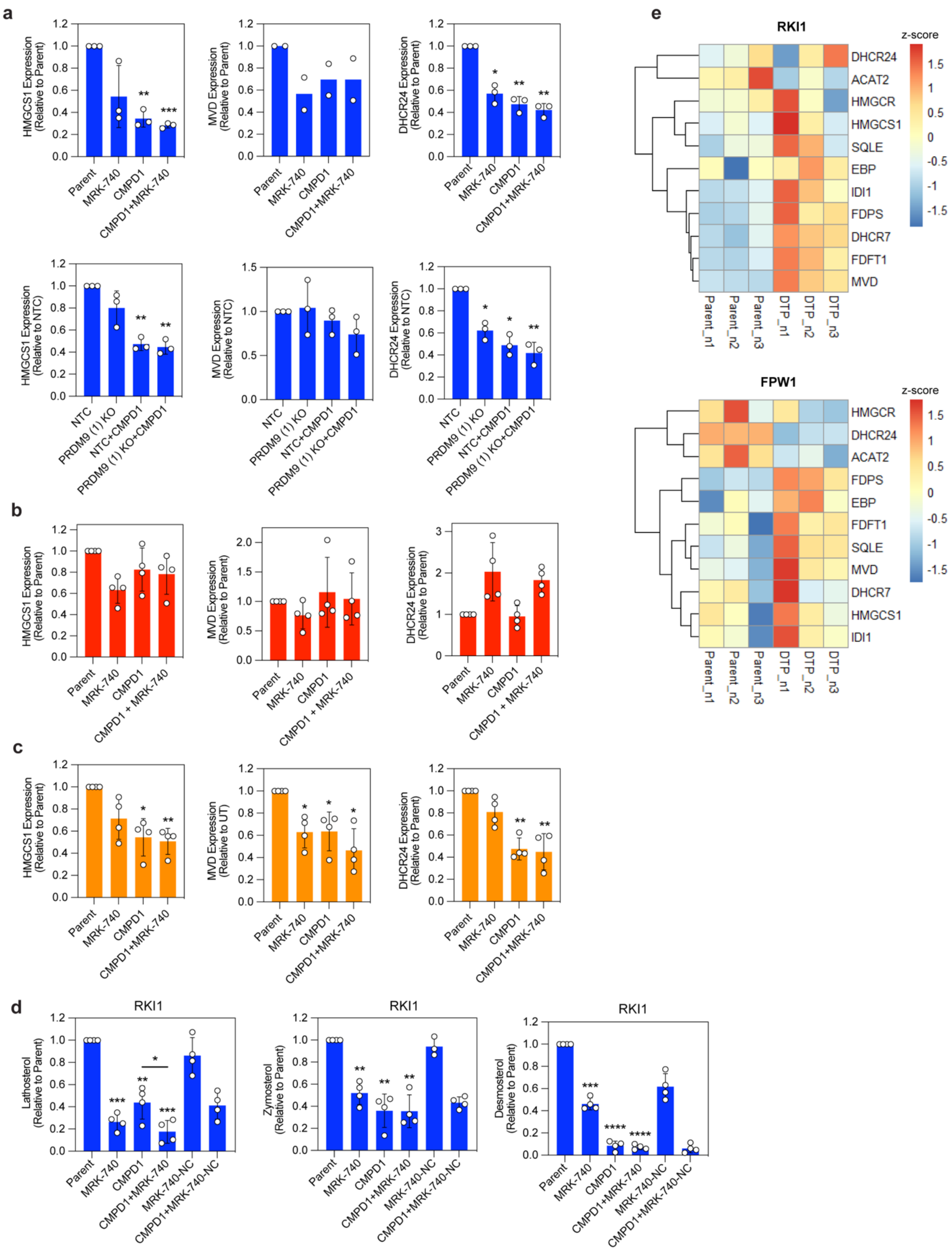

Supplementary Figure 6. a Quantification of HMGCS1, MVD, DHCR24 normalised to GAPDH in RKI1 cells treated with CMPD1 (10 µM, 3 days) ± MRK-740 (3 µM) (top row). Quantification of HMGCS1, MVD and DHCR24 in CMPD1 (10 µM, 3 days) treated RKI1 cells transduced with NTC sgRNA (NTC) or PRDM9 sgRNA (PRDM9 KO) (lower row). Related to immunoblots in Figure 7b. Data are mean (n = 2 biological replicates) for MVD; and mean ± SD (n = 3 biological replicates) for HMGCS1 and DHCR24. One sample t-test: * indicates p (two tailed) < 0.05; **indicates p (two tailed) < 0.01, *** indicates p (two tailed) < 0.001. **b** Quantification of HMGCS1, MVD, DHCR24 normalised to GAPDH in FPW1 cells treated with CMPD1 (10 µM, 6 days) ± MRK-740 (5 µM). Related to immunoblots in Figure 7c. Data are mean ± SD (n = 4 biological replicates). **c** Quantification of HMGCS1, MVD, DHCR24 normalised to GAPDH in MMK1 cells treated with CMPD1 (10 µM, 3 days) ± MRK-740 (5 µM). Related to immunoblots in Figure 7d. Data are mean ± SD (n = 4 biological replicates). One sample t-test: * indicates p (two tailed) < 0.05; **indicates p (two tailed) < 0.01. **d** Cholesterol intermediates in RKI1 cells treated with CMPD1 (10 µM, 3 days) ± MRK-740 (3 µM)/MRK-740-NC (3µM). Data are mean ± SD (n = 4 biological replicates). One sample t-test: * indicates p (two tailed) < 0.05; **indicates p (two tailed) < 0.01, *** indicates p (two tailed) < 0.001, **** indicates p (two tailed) < 0.0001. **e** Heatmap of cholesterol genes in CMPD1 (25 µM, 14 days) derived drug-tolerant persister (DTP) cells compared to parent cells (RNA sequencing of n = 3 biological replicates). Source data are provided as a Source Data file.

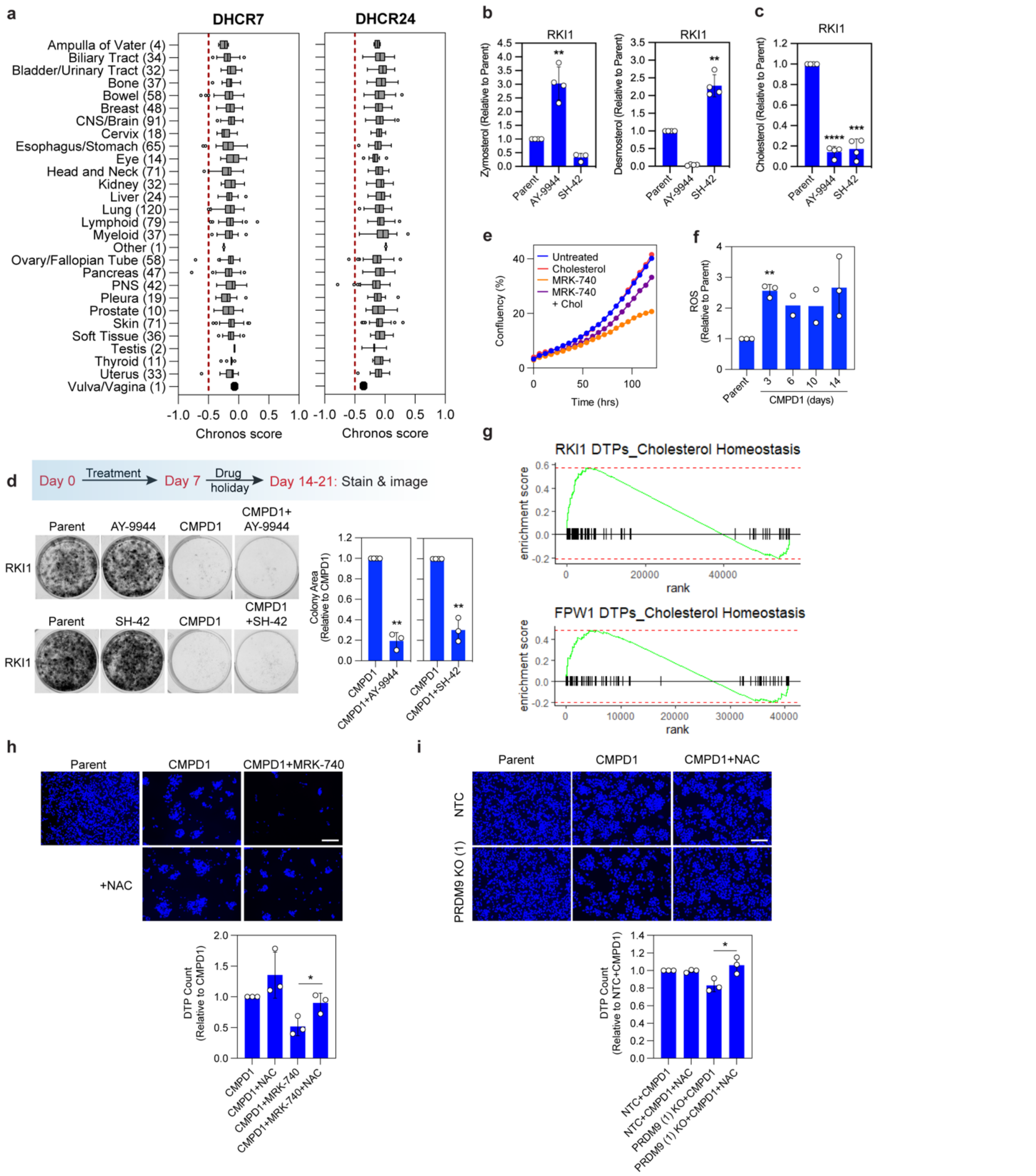

Supplementary Figure 7. a Chronos scores for DHCR7 and DHCR24 knockout in 1,095 cancer cell lines (23Q2[+] Score dataset, DepMap). Box plots display the mean as the centre line, the box representing the 25th – 75th percentiles, and the whiskers extending to 1.5x the interquartile range (IQR) beyond the box limits. Outliers are shown as individual data points. **b - c** Desmosterol, zymosterol and cholesterol in RKI1 cells treated with AY-9944 (1 µM, 3 days) or SH-42 (1 µM, 3 days). Data are mean ± SD (n = 4 biological replicates). One sample t-test: ** indicates p (two tailed) = 0.0067, p (two tailed) = 0.0038 for AY-9944 and SH-42, respectively. **d** Representative images and quantification RKI1 colonies treated with CMPD1 (25 µM) ± AY-9944 or SH-42 (1 µM) for 7 days, followed by recovery in drug-free media. Data are mean ± SD (n = 3 biological replicates). One sample t-test: ** indicates p (two tailed) = 0.0032, p (two tailed) = 0.0090 for CMPD1+AY-9944 and CMPD1+SH-42, respectively. **e** Incucyte imaging of RKI1 cells treated with MRK-740 (3 µM) ± cholesterol (5 µg/mL). Data is mean of n = 3 biological replicates. **f** ROS quantification in RKI1 cells treated with CMPD1 (10 µM). Data are mean (n = 2 biological replicates) for day 6 and 10; and mean ± SD (n = 3 biological replicates) for day 3 and 14. One sample t-test: ** indicates p (two tailed) = 0.0056. **g** Barcode enrichment plot for cholesterol homeostasis hallmark in CMPD1 (25 µM, 14 days) derived DTP cells compared to parent cells (via RNA sequencing of n = 3 biological replicates). Genes were ranked by log2 Fold-Change in descending order. **h** DAPI stained images and quantification of DTPs in RKI1 cells treated with CMPD1 (25 µM, 7 days) ± MRK-740 (3 µM) ± N-acetyl-cysteine (NAC, 1 mM). Data are mean ± SD (n = 4 biological replicates). Unpaired t-test: * indicates p (two tailed) = 0.0394. Scale bar 100 µm. **i** DAPI-stained images and quantification of DTPs in RKI1 cells transduced with NTC sgRNA or PRDM9 (1) sgRNA, treated with CMPD1 (25 µM, 7 days) ± NAC (1 mM). Data are mean ± SD (n = 4 biological replicates). Unpaired t-test: * indicates p (two tailed) = 0.0281. Scale bar 100 µm. Source data are provided as a Source Data file.

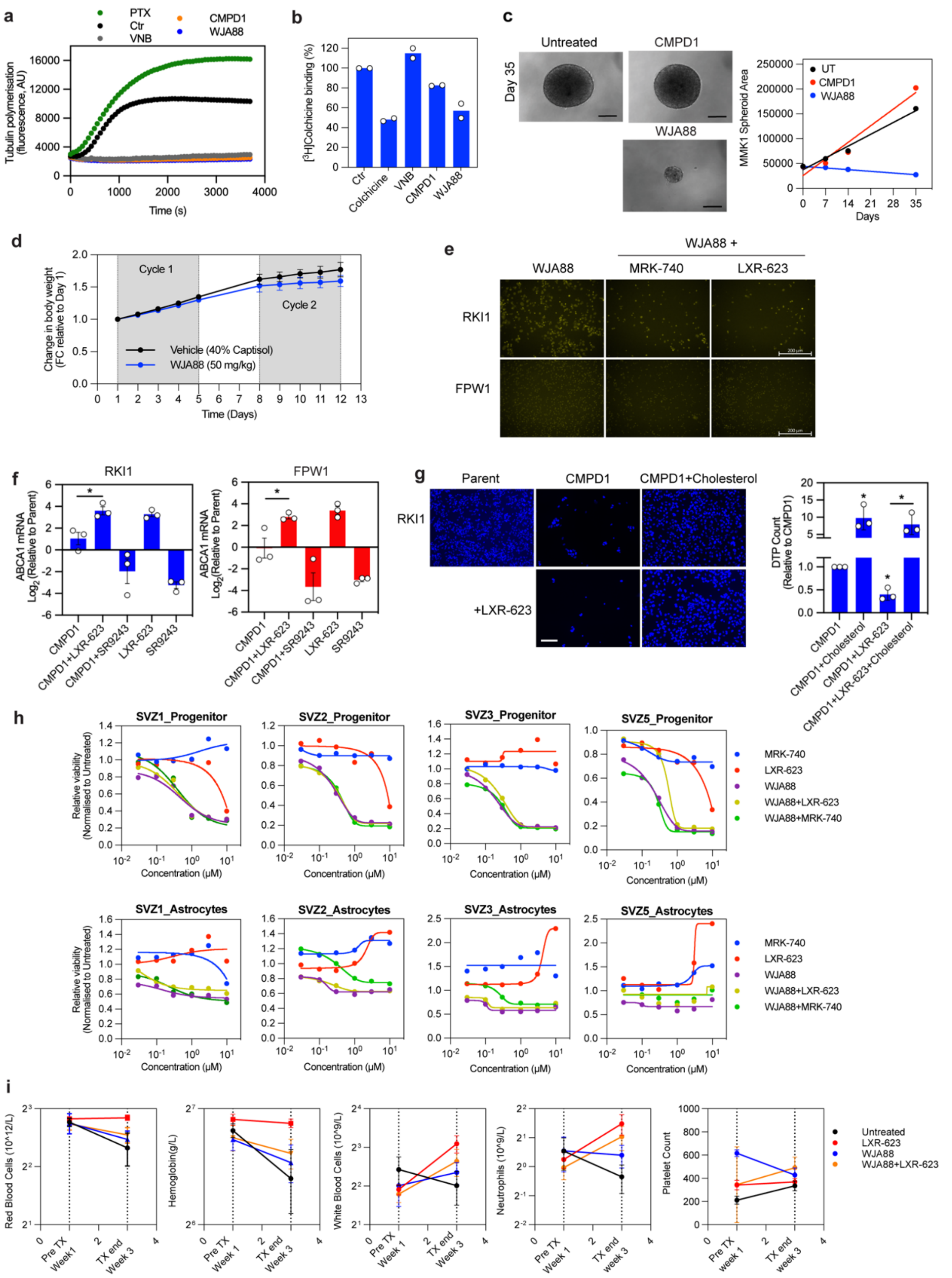

Supplementary Figure 8. a Kinetics of tubulin polymerisation in the presence of paclitaxel (PTX, 3 µM), vinblastine (VNB, 3 µM), CMPD1 (50 µM) and WJA88 (50 µM). Data are mean (n = 3 biological replicates). **b** Binding of $[^3H]$colchicine in the presence of colchicine, vinblastine, CMPD1 and WJA88 (all 10 µM). Data are mean (n = 2 biological replicates). **c** Images and quantification of MMK1 spheroids growth treated with CMPD1 (5 µM) and WJA88 (5 µM). Data are mean of 2-3 spheroids per treatment. Scale bar 100 nm. **d** Weight change of NPP-tdTomato tumour bearing mice over the treatment course. Shaded area indicates period of daily IP injection of vehicle or WJA88. Data are mean ± SD (n = 5 mice per treatment). **e** Representative nuclear-ID red stain images (pseudo-coloured yellow) of RKI1 and FPW1 drug-tolerant persister (DTP) cells surviving WJA88 (25 µM, 14 days) ± MRK-740 (3 µM) or LXR-623 (1 µM). Relates to quantification data in Figure 8g (n = 4 biological replicates). Scale bar 200 µm. **f** ABCA1 mRNA expression in RKI1 and FPW1 cells treated with CMPD1 (25 µM, 3 days) ± LXR-623 (1 µM) or SR9243 (1 µM). Data are mean ± SD (n = 3 biological replicates). Unpaired t-test: * indicates p (two tailed) = 0.0426, p (two tailed) = 0.0162 for RKI1 and FPW1, respectively. **g** Representative DAPI-stained images and quantification of RKI1 cells treated with CMPD1 (25 µM, 14 days) ± LXR-623 (1 µM) ± cholesterol (5% w/w). Data are mean ± SD (n = 3 biological replicates. One sample t-test between co-treatment and CMPD1: * indicates p (two tailed) = 0.0473, p (two tailed) = 0.0151 for CMPD1+Chol and CMPD1+LXR-623, respectively. Unpaired t-test between co-treatments: * indicates p (two tailed) = 0.0119. Scale bar 100 µm. **h** Cell viability of mice-derive neural progenitor cells and astrocytes treated with WJA88 (25 µM, 5 days) ± MRK-740 (3 µM) ± LXR-623 (1 µM). **i** Blood counts (n = 3 mice per treatment) measured using a Forcyte Hematology Analyzer. Source data are provided as a Source Data file.

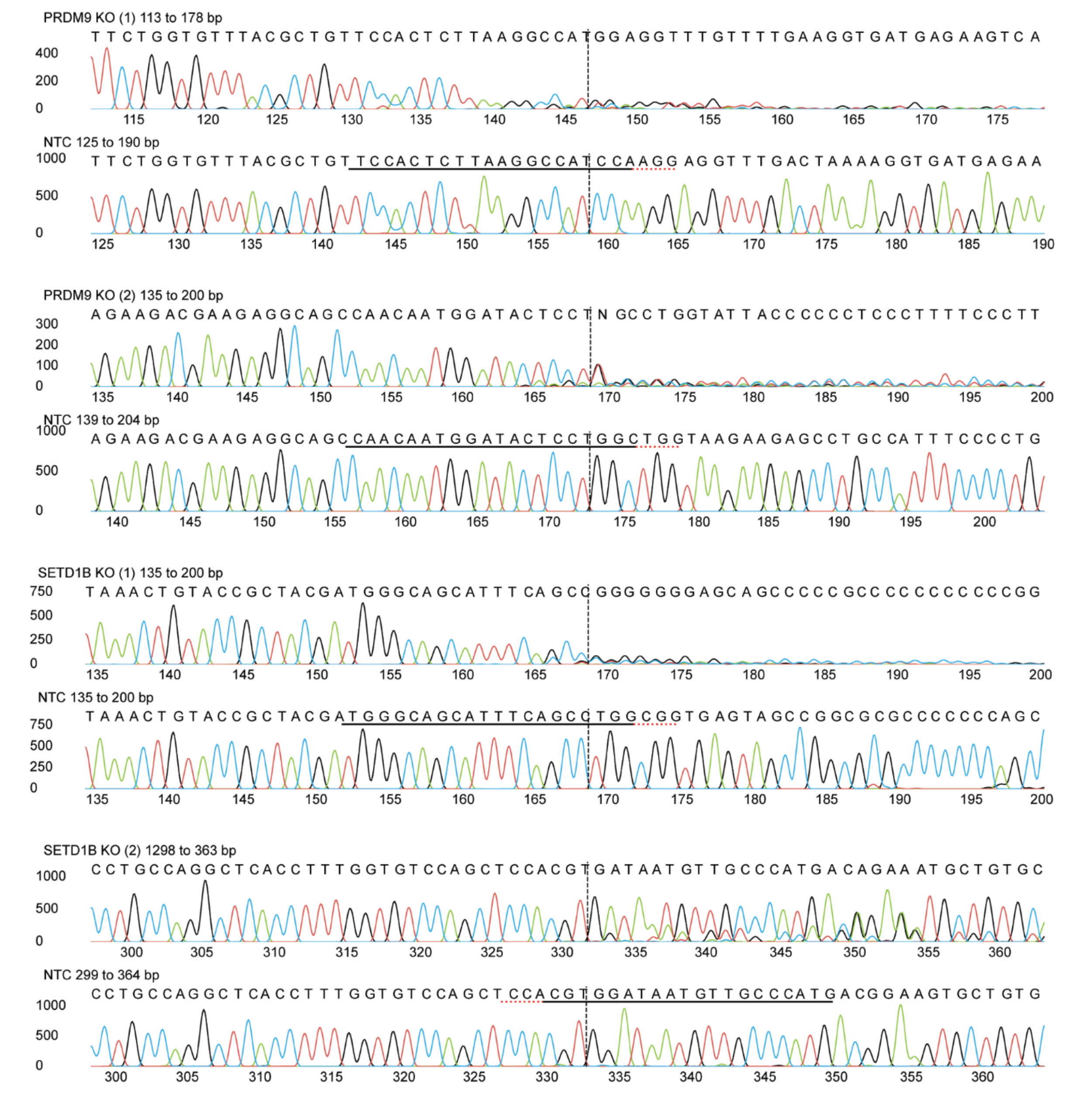

Supplementary Figure 9. Sanger sequencing of genomic DNA at the CRISPR/Cas9-targeted knockout site for each knockout cell line. Chromatograms show the sequencing traces aligned to the reference sequence, confirming insertions/deletions at the target locus.

Supplementary Table 1. Cellular efficacy (EC_{50}; values are mean of indicated biological replicates) of CMPD1, analogues **1 - 17** and WJA88 in A172 cells.

**Compound**	**Structure**	**EC_{50} (µM)**
CMPD1	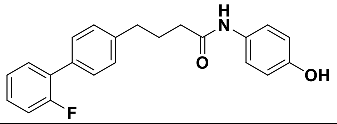	0.26<br>(n = 7)
**2**	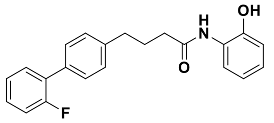	>50<br>(n = 3)
**3**	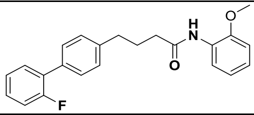	>50<br>(n = 3)
**4**	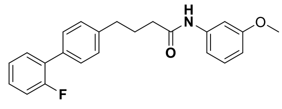	5.1<br>(n = 2)
**5**	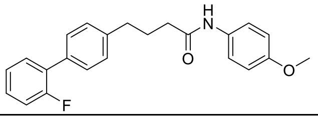	7.9<br>(n = 2)
**6**	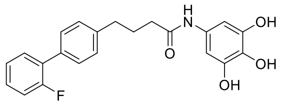	>50<br>(n = 3)
**7**	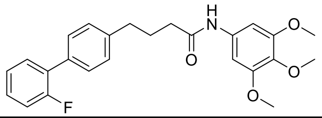	>10<br>(n = 3)
**8**	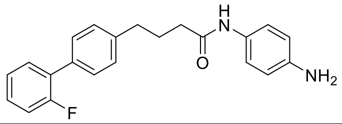	1.5<br>(n = 2)
**9**	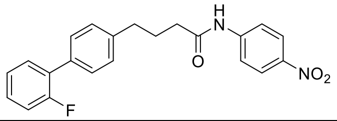	>10<br>(n = 3)
**10**	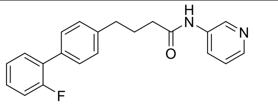	0.36<br>(n = 3)
**11**	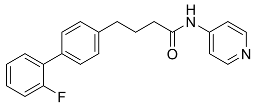	>50<br>(n = 3)
**12**	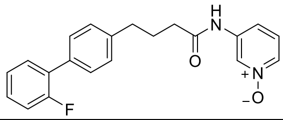	>50<br>(n = 3)
**13**	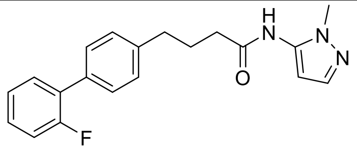	2.5<br>(n = 2)
**14**	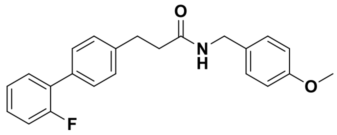	>50<br>(n = 3)

**15**	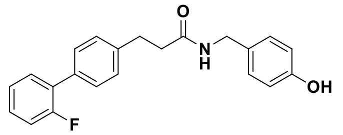	3.3<br>(n = 2)
**16**	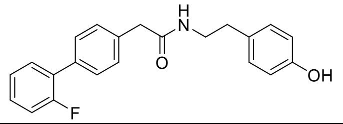	>10<br>(n = 3)
**17**	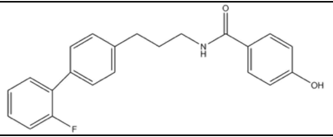	3.5<br>(n = 2)
**WJA88**	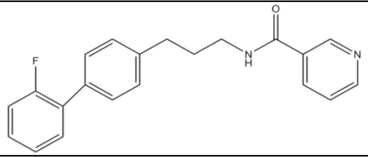	0.15<br>(n =2)

Supplementary Table 2. Cellular efficacy (EC_{50}; values are mean of indicated biological replicates) of CMPD1, analogue **10** and WJA88 in glioblastoma stem cell lines.

Cell line	CMPD1 EC_{50} (µM)	Analogue 10 EC_{50} (µM)	WJA88 EC_{50} (µM)
**MMK1**	0.85 (n = 3)	1.6 (n = 3)	0.31 (n = 3)
**HW1**	0.60 (n = 3)	0.80 (n = 3)	0.76 (n = 3)
**SB2**	0.28 (n = 2)	0.32 (n = 2)	0.33 (n = 2)
**PB1**	0.51 (n = 3)	0.56 (n = 3)	0.39 (n = 3)
**RN1**	1.05 (n = 2)	1.50 (n = 2)	0.80 (n = 2)
**WK1**	0.61 (n = 3)	0.83 (n = 3)	0.66 (n = 3)
**RKI1**	0.96 (n = 3)	0.98 (n = 3)	0.79 (n = 3)

Supplementary Table 3. Pharmacokinetic parameters of WJA88 after single 50 mg/kg dose administered intraperitoneally to male CD-1 mice (mean of n = 3 mice).

	WJA88
**C_{max} (ng/mL)**	7907
**T_{max} (h)**	0.25
**$T_{1/2}$ (h)**	1.19
**T_{last} (h)**	8.00
**AUC_{0-last} (ng.h/mL)**	4262
**AUC_{0-inf} (ng.h/mL)**	4271
**MRT_{0-last} (h)**	0.883
**MRT_{0-inf} (h)**	0.905
**AUC_{Extra} (%)**	0.263
**$AUMC_{Extra}$ (%)**	3.00

SUPPLEMENATRY REFERENCES

1. Wu Y*, et al.* Glioblastoma epigenome profiling identifies SOX10 as a master regulator of molecular tumour subtype. *Nature Communications* **11**, 6434 (2020).
