# Supplementary material for: Histone methyltransferase PRDM9 promotes survival of drug-tolerant persister cells in glioblastoma
Source: Nat Commun. 2025 Dec 15;16:10905. doi: 10.1038/s41467-025-65888-5 (PMC12705669; doi:10.1038/s41467-025-65888-5)
Supplement: Supplementary file 2 — Description of Additional Supplementary Information [file 41467_2025_65888_MOESM2_ESM.pdf]

Supplementary Data 1. Total H3K4me3 ChIP-seq peaks in RKI1 parent cells. Genomic regions (hg38) (chromosome, start coordinate, end coordinate) of H3K4me3 ChIP-seq peaks in RKI1 parent cells. Peaks were calculated using MACS2.0 package with a q-value cutoff of 0.05 and mfold between 5 and 50.

Supplementary Data 2. PRDM9 motif hg38. Genomic regions (hg38) (chromosome, start co-ordinate, end co-ordinate) of PRDM9 motif occurrences in the hg38 genome ($p<0.0001$) were identified using the human PRDM9 consensus motif (JASPAR ID: MA1723.1) with FIMO package (Meme Suite 5.5.5).

Supplementary Data 3. hg38 PRDM9 motif overlaps with H3K4me3 in RKI1 parent cells. Genomic regions (hg38) (chromosome, start co-ordinate, end co-ordinate) of overlapping high confidence PRDM9 motif (Supplementary Data 2) sequences with total H3K4me3 ChIP-seq peaks, in RKI1 Parent cells (Supplementary Data 1).

Supplementary Data 4. Total H3K4me3 ChIP-seq peaks in untreated RKI1 NTC cells. Genomic regions (hg38) (chromosome, start coordinate, end coordinate) of H3K4me3 ChIP-seq peaks in untreated RKI1 NTC cells. Peaks were calculated using MACS2.0 package with a q-value cutoff of 0.05 and mfold between 5 and 50.

Supplementary Data 5. hg38 PRDM9 motif overlaps with H3K4me3 in untreated RKI1 NTC cells. Genomic regions (hg38) (chromosome, start co-ordinate, end co-ordinate) of overlapping high confidence PRDM9 motif (Supplementary Data 2) sequences with total H3K4me3 ChIP-seq peaks, in RKI1 NTC untreated cells (Supplementary Data 4).

Supplementary Data 6. hg19 PRDM9 motif overlaps with H3K4me3 ChIP-seq peaks in AK124 glioblastoma tumour. Genomic regions (hg19) (chromosome, start co-ordinate, end co-ordinate) of overlapping high confidence PRDM9 motif sequences (calculated with FIMO package $p<0.0001$, human PRDM9 JASPAR ID: MA1723.1, on hg19 genome) with total H3K4me3 ChIP-seq peaks (hg19), in AK124 patient glioblastoma tumour.

Supplementary Data 7. hg19 PRDM9 motif overlaps with H3K4me3 ChIP-seq peaks in AK231 glioblastoma tumour. Genomic regions (hg19) (chromosome, start co-ordinate, end co-ordinate) of overlapping high confidence PRDM9 motif sequences (calculated with FIMO package $p<0.0001$, human PRDM9 JASPAR ID: MA1723.1, on hg19 genome) with total H3K4me3 ChIP-seq peaks (hg19), in AK231 patient glioblastoma tumour.
