# Supplementary material for: Histone methyltransferase PRDM9 promotes survival of drug-tolerant persister cells in glioblastoma
Source: Nat Commun. 2025 Dec 15;16:10905. doi: 10.1038/s41467-025-65888-5 (PMC12705669; doi:10.1038/s41467-025-65888-5)
Supplement: Supplementary file 4 — Reporting Summary [file 41467_2025_65888_MOESM4_ESM.pdf]

nature portfolio

Corresponding author(s): Lenka Munoz

Last updated by author(s): Oct 24, 2025

Reporting Summary

Nature Portfolio wishes to improve the reproducibility of the work that we publish. This form provides structure for consistency and transparency in reporting. For further information on Nature Portfolio policies, see our Editorial Policies and the Editorial Policy Checklist.

Statistics

For all statistical analyses, confirm that the following items are present in the figure legend, table legend, main text, or Methods section.

n/a	Confirmed	
☐	☒	The exact sample size (*n*) for each experimental group/condition, given as a discrete number and unit of measurement
☐	☒	A statement on whether measurements were taken from distinct samples or whether the same sample was measured repeatedly
☐	☒	The statistical test(s) used AND whether they are one- or two-sided<br>*Only common tests should be described solely by name; describe more complex techniques in the Methods section.*
☒	☐	A description of all covariates tested
☐	☒	A description of any assumptions or corrections, such as tests of normality and adjustment for multiple comparisons
☐	☒	A full description of the statistical parameters including central tendency (e.g. means) or other basic estimates (e.g. regression coefficient) AND variation (e.g. standard deviation) or associated estimates of uncertainty (e.g. confidence intervals)
☐	☒	For null hypothesis testing, the test statistic (e.g. *F*, *t*, *r*) with confidence intervals, effect sizes, degrees of freedom and *P* value noted<br>*Give P values as exact values whenever suitable.*
☒	☐	For Bayesian analysis, information on the choice of priors and Markov chain Monte Carlo settings
☒	☐	For hierarchical and complex designs, identification of the appropriate level for tests and full reporting of outcomes
☐	☒	Estimates of effect sizes (e.g. Cohen's *d*, Pearson's *r*), indicating how they were calculated

Our web collection on statistics for biologists contains articles on many of the points above.

Software and code

Policy information about availability of computer code

Data collection	Illumina Nextseq1000/2000 Control Software Suite v1.7.1<br>Illumina DRAGEN RNA (v4.2.7)<br>Illumina DRAGEN BCL Convert (v4.2.7)<br>Agilent MassHunter<br>BioRad Image Lab v6.1<br>Roche LightCycler® 480 Software<br>Zeiss ZEN lite<br>Sartorius Incucyte 2022A<br>BD FACSDiva software<br>Tecan Magellan
Data analysis	GraphPad PRISM v10.1.2<br>Python v3.11 Command Line: Deeptools 2.0, pyGenomeTracks, MEME Suite 5.5.7<br>RStudio: in built functions, ggplot2, chipseeker, pheatmap, biomaRt, clusterProfiler, org.Hs.eg.db, GenomicRanges, EnsDb.Hsapiens.v86, AnnotationDbi, Combat, dplyr.<br>Mascot v2.4<br>Skyline v20.1<br>Thermofisher Scientific TraceFinder v4.1<br>FlowJo v10.8.1<br>Synthego ICE Analysis tool

Venny
ImageJ
ImageJ Fiji

For manuscripts utilizing custom algorithms or software that are central to the research but not yet described in published literature, software must be made available to editors and reviewers. We strongly encourage code deposition in a community repository (e.g. GitHub). See the Nature Portfolio guidelines for submitting code & software for further information.

Data

Policy information about availability of data

All manuscripts must include a data availability statement. This statement should provide the following information, where applicable:

- Accession codes, unique identifiers, or web links for publicly available datasets
- A description of any restrictions on data availability
- For clinical datasets or third party data, please ensure that the statement adheres to our policy

The source data file containing raw data for all figures and uncropped scans of western blots are provided with this paper. All next generation RNAsequencing and ChIPsequencing datasets have been deposited into Gene Expression Omnibus (GEO) under the accession number GSE279066 (URL: https://www.ncbi.nlm.nih.gov/geo/query/acc.cgi?acc=GSE279066). The Heidelberg glioblastoma tumour ChIPseq and RNAseq dataset used for this paper is under accession number GSE121723 (URL: https://www.ncbi.nlm.nih.gov/geo/query/acc.cgi?acc=GSE121723) (Refence 39). The mass spectrometry proteomics raw data files have been deposited to the ProteomeXchange Consortium via the PRIDE partner repository with the dataset identifier PXD050643. Lipidomics raw data data (Study ID ST004262; DatatrackID:6506) have been deposited in the Metabolomics Workbench (Reference 74) and made publicly available (URL: http://dx.doi.org/10.21228/M82R9H). The remaining data are available within the Article, Supplementary Data, Supplementary Information and Source Data files.

Research involving human participants, their data, or biological material

Policy information about studies with human participants or human data. See also policy information about sex, gender (identity/presentation), and sexual orientation and race, ethnicity and racism.

Reporting on sex and gender	Population statement from the authors of GSE121723 (note that 19/60 samples were analysed, that contained the relevant ChIPseq data for our analysis goals):<br><br>"The patient cohort consisted of 32 males and 28 females with an average age of 52.5±11 years (mean and standard deviation)."<br><br>Wu Y, Fletcher M, Gu Z, Wang Q et al. Glioblastoma epigenome profiling identifies SOX10 as a master regulator of molecular tumour subtype. Nat Commun 2020 Dec 18;11(1):6434. PMID: 33339831<br><br>The findings of this cohort therefore apply to both sexes. Explicit, individual level data on sex, is not provided, although it can be indirectly determined by the presence of a Y chromosome in their sequencing data sets.
Reporting on race, ethnicity, or other socially relevant groupings	There was no reporting, by the authors of GSE121723, to this effect, and such groupings are not relevant to our study.
Population characteristics	All patients received the standard of care therapy for Glioblastoma: a combination of Radiotherapy and Temozolamide. Human glioblastoma H3K4me3 and H3K36me3 ChIPseq and RNAseq data was sourced from GSE121723. Processed data files, for 19 glioblastoma patients from this data set, were re-analysed in accordance with the goals of this study.
Recruitment	The authors of GSE121723 undertook a retrospective study.
Ethics oversight	Ethics statement from the authors of GSE121723:<br><br>"Snap-frozen primary glioblastoma tumour samples and clinical data were collected at the time of primary diagnosis between 1994 and 2011 at the Burdenko Neurosurgery Institute (Moscow, Russia). Informed consent was obtained from all patients. Use of the material and clinical data for this study was approved by the ethics board at the Burdenko Neurosurgery Institute (Moscow, Russia)."<br><br>Wu Y, Fletcher M, Gu Z, Wang Q et al. Glioblastoma epigenome profiling identifies SOX10 as a master regulator of molecular tumour subtype. Nat Commun 2020 Dec 18;11(1):6434. PMID: 33339831

Note that full information on the approval of the study protocol must also be provided in the manuscript.

Field-specific reporting

Please select the one below that is the best fit for your research. If you are not sure, read the appropriate sections before making your selection.

[x] Life sciences [ ] Behavioural & social sciences [ ] Ecological, evolutionary & environmental sciences

For a reference copy of the document with all sections, see nature.com/documents/nr-reporting-summary-flat.pdf

Life sciences study design

All studies must disclose on these points even when the disclosure is negative.

Sample size	Generally, for in vitro experiments, n=3 biological replicates was used as a minimum guideline. Provided a high confidence statistical trend emerged from three repeats, attributed to biology, and to the exclusion of the possibility of technical variation, the authors were satisfied to present the results, and conduct statistical testing. If additional repeats were required, it was conducted until a high confidence trend was observed. For sequencing experiments, the ENCODE guidelines were adhered to. For RNAseq, all experiments were designed with n=3 biological repeats, per cell line. For ChIPseq, n=2 biological repeats were analysed, according to the ENCODE guidelines, given the qualitative nature of assessing ChIPseq replicates individually. For protein liquid chromatography-mass spectrometry, n=3 biological replicates were conducted, per specified cell line. For lipid LC-MS, n=3 biological replicates were conducted. For Gas Chromatograph-Mass Spectrometry of Cholesterol and Sterol precursors, n=4 biological replicates were conducted. For electroporation model of high grade glioma, n=5 CD-1 mice was utilised per treatment arm. For GBM6 orthotopic xenograft experiments, n=8 animals BALB/c mice per treatment condition was utilised to calculate survival, weight over time. Blood counts were conducted with n=3 mice, per treatment condition.
Data exclusions	In an RNA-seq experiment (GEO superseries: GSE279066, GEO subseries GSE279420), a repeat (repeat 2/3) of RKI1 Non-Targeted Control cells treated with CMPD1 (Sample GSM8569627) did not cluster according to condition (Principal Component Analysis) with the other two repeats. It was subsequently excluded from down-stream analysis. The data was uploaded to GEO (GEO superseries: GSE279066, GEO subseries GSE279420).
Replication	n>=3 biological replicates for in vitro cellular experiments unless otherwise specified. Replication of data was successful, except for the biological replicate 2/3 of RKI1 Non-targeted control cells which was excluded as described above. For ChIPseq for which n=2 biological replicates were analysed, per ENCODE guidelines. The in vivo animal experiments were performed with at least n=5 (brain electroporation glioma model) or n=8 (orthotopic xenograft model) mice per treatment group.
Randomization	Cells and animals were randomly grouped for the experiments.
Blinding	For in vitro experiments, blinding was not possible. Measurements were always taken by instruments, and therefore, experimental values were never subject to experimenter bias. Blinding was done for the analysis of the brain electroporation glioma study.

Reporting for specific materials, systems and methods

We require information from authors about some types of materials, experimental systems and methods used in many studies. Here, indicate whether each material, system or method listed is relevant to your study. If you are not sure if a list item applies to your research, read the appropriate section before selecting a response.

Materials & experimental systems

n/a	Involved in the study	
☐	☒	Antibodies
☐	☒	Eukaryotic cell lines
☒	☐	Palaeontology and archaeology
☐	☒	Animals and other organisms
☒	☐	Clinical data
☒	☐	Dual use research of concern
☒	☐	Plants

Methods

n/a	Involved in the study	
☐	☒	ChIP-seq
☐	☒	Flow cytometry
☒	☐	MRI-based neuroimaging

Antibodies

Antibodies used

Application Western Blot:
(all Cell Signalling Technologies): GAPDH (Cat# 97166, 1:2000 dilution for western blot), H3K4me1 (Cat# 5326, 1:1000 dilution for western blot), H3K4me2 (Cat# 9725, 1:1000 dilution for western blot), H3K4me3 (Cat# 9751S, 1:1000 dilution for western blot), H3K27me1 (Cat# 84932, 1:1000 dilution for western blot), H3K27me3 (Cat# 9733, 1:1000 dilution for western blot), Histone H3 (Cat# 4499, 1:2000 dilution for western blot), Cleaved Histone H3 (Thr22) (Cat# 12576, 1:1000 dilution for western blot), DHCR24 (Cat# 2033, 1:1000 dilution for western blot), HMGCS1 (Cat# 36877, 1:1000 dilution for western blot), SET1B (Cat# 44922, 1:1000 dilution for western blot), Rabbit IgG HRP-linked (Cat# 7074, 1:2000 dilution for western blot) and Mouse IgG HRP-linked (Cat# 7076, 1:2000 dilution for western blot). ABCAM antibodies against: H3K4me3 (Cat# ab12209, 1:1000 dilution for western blot), H3K9me1 (Cat# ab9045, 1:1000 dilution for western blot), H3K9me2 (Cat# ab1220, 1:1000 dilution for western blot), H3K9me3 (Cat# ab8898, 1:1000 dilution for western blot), H3K27me2 (Cat# ab24684, 1:1000 dilution for western blot), H3K36me1 (Cat# ab9048, 1:1000 dilution for western blot), H3K36me2 (Cat# ab9049, 1:1000 dilution for western blot), H3K36me3 (Cat# ab9050, 1:1000 dilution for western blot), Histone H3.3 (Cat# ab176840, 1:1000 dilution for western blot) and MVD (Cat# ab129061, 1:1000 dilution for western blot), Histone H3.1/3.2 (MerckMillipore Cat# ABE154, 1:1000 dilution for western blot), PRDM9 (ThermoFisher Scientific Cat# MA5-51196, 1:1000 dilution for western blot).

Application ChIPseq:
Cell Signalling Technnology: H3K4me3 (Cat# 9751S, 1:50 for ChIP-seq).

Application: Immunofluorescence:

BD Pharmingen: Ki67 (Cat# 550609, 1:100 dilution for immuno-fluorescence). Jackson ImmunoResearch: Anti-mouse 647 secondary antibody (Cat# #715-606-151, 1:500 dilution for immunofluorescence).

Validation

Cell Signalling Technology:

GAPDH (Cat# 97166, 1:2000 dilution for western blot): Source/Purification: Monoclonal antibody is produced by immunizing animals with a synthetic peptide corresponding to residues near the amino terminus of human GAPDH protein. Validation (Specific to applicable techniques in this study): Western blot analysis of extracts from various cell lines using GAPDH (D4C6R) Mouse mAb. Single band at correct molecular weight.

H3K4me1 (Cat# 5326, 1:1000 dilution for western blot): https://www.antibodyregistry.org/AB_10695148

H3K4me2 (Cat# 9725, 1:1000 dilution for western blot): https://www.antibodyregistry.org/AB_10205451

H3K4me3 (Cat# 9751S, 1:1000 dilution for western blot, 1:50 for ChIP-seq): https://www.antibodyregistry.org/AB_2616028

H3K27me1 (Cat# 84932, 1:1000 dilution for western blot): https://www.antibodyregistry.org/AB_2800043

H3K27me3 (Cat# 9733, 1:1000 dilution for western blot): https://www.antibodyregistry.org/AB_2616029

Histone H3 (Cat# 4499, 1:2000 dilution for western blot): https://www.antibodyregistry.org/AB_10544537

Cleaved Histone H3 (Thr22) (Cat# 12576, 1:1000 dilution for western blot): https://www.antibodyregistry.org/AB_2797961

DHCR24 (Cat# 2033, 1:1000 dilution for western blot): https://www.antibodyregistry.org/AB_2091448

HMGCS1 (Cat# 36877, 1:1000 dilution for western blot): https://www.antibodyregistry.org/AB_2799107

SET1B (Cat# 44922, 1:1000 dilution for western blot): https://www.antibodyregistry.org/AB_2799275

Rabbit IgG HRP-linked (Cat# 7074, 1:2000 dilution for western blot): https://www.antibodyregistry.org/AB_2099233

Mouse IgG HRP-linked (Cat# 7076, 1:2000 dilution for western blot): https://www.antibodyregistry.org/AB_330924

ABCAM:

H3K4me3 (Cat# ab12209, 1:1000 dilution for western blot): https://www.antibodyregistry.org/AB_442957

H3K9me1 (Cat# ab9045, 1:1000 dilution for western blot): https://www.antibodyregistry.org/AB_306963

H3K9me2 (Cat# ab1220, 1:1000 dilution for western blot): https://www.antibodyregistry.org/AB_449854

H3K9me3 (Cat# ab8898, 1:1000 dilution for western blot): https://www.antibodyregistry.org/AB_306848

H3K27me2 (Cat# ab24684, 1:1000 dilution for western blot): https://www.antibodyregistry.org/AB_448222

H3K36me1 (Cat# ab9048, 1:1000 dilution for western blot): https://www.antibodyregistry.org/AB_306964

H3K36me2 (Cat# ab9049, 1:1000 dilution for western blot): https://www.antibodyregistry.org/AB_1280939

H3K36me3 (Cat# ab9050, 1:1000 dilution for western blot): https://www.antibodyregistry.org/AB_306966

Histone H3.3 (Cat# ab176840, 1:1000 dilution for western blot): https://www.antibodyregistry.org/AB_2715502

MVD (Cat# ab129061, 1:1000 dilution for western blot): https://www.antibodyregistry.org/AB_11145667

MerckMillipore:

Histone H3.1/3.2 (Cat# ABE154, 1:1000 dilution for western blot): https://www.antibodyregistry.org/AB_2811170

ThermoFisher Scientific:

PRDM9 (Cat# MA5-51196, 1:1000 dilution for western blot): https://www.antibodyregistry.org/AB_3094253

BD Pharmingen:

Ki67 (Cat# 550609, 1:100 dilution for immuno-fluorescence): https://www.antibodyregistry.org/AB_393778

Jackson ImmunoResearch:

Anti-mouse 647 secondary antibody (Cat# #715-606-151, 1:500 dilution for immuno-fluorescence): https://www.antibodyregistry.org/AB_2340866

Eukaryotic cell lines

Policy information about cell lines and Sex and Gender in Research

Cell line source(s)	QIMR Berghofer QCell Glioblastoma Stem Cell Lines:<br><br>Patient Age_(years) Gender Tumour_type Tumour_site<br>FPW1 68 Male Primary GBM Right temporal<br>HW1 54 Female Primary GBM Right frontal parietal<br>JK2 75 Male Primary GBM Right frontal<br>MMK1 80 Female Primary GBM Right temporal<br>RKI1 57 Female Primary GBM Left temporal<br>SB2b 48 Male Recurrent GBM Right parietal<br>WK1 77 Male Primary GBM Right parietal occipital<br><br>GBM6 glioblastoma cells (EGFR/EGFRvIII over-expression) were obtained from Paul Mischel, Ludwig Institute of Cancer Research, USA.<br><br>Primary neural stem cell cultures and their differentiated astrocytes were derived from subventricular zone dissections of C57BL/6 mouse brains.
Authentication	No authentication was performed for cell lines.
Mycoplasma contamination	All cell lines used in this study, were routinely tested for Mycoplasma contamination. All cell lines were bi-monthly sent for institutional testing, using Lonza MycoAlert™ PLUS Mycoplasma Detection Kit.
Commonly misidentified lines<br>(See ICLAC register)	No commonly misidentified cell lines were used.

Animals and other research organisms

Policy information about studies involving animals; ARRIVE guidelines recommended for reporting animal research, and Sex and Gender in Research

Laboratory animals	CD-1 mice were used for electroporation model of high grade glioma. Experimental treatments began three weeks post electroporation (this occurred on day 2 of the mice pup's life). Both female and male mice were utilised. Female BALB/c Nude mice were used for orthotopic GBM6 cell injection into the cortex. Female and male C57bl/6 mice were used to derive neural stem cells.
Wild animals	No wild animals were used in this study.
Reporting on sex	Sex was not considered, animals were randomly grouped into treatments.
Field-collected samples	No field-collected samples were used in this study.
Ethics oversight	Animal studies were performed under the Telethon Kids Institute Animal Ethics and QIMR Berghofer rules and regulations, all protocols were approved by relevant institution.

Note that full information on the approval of the study protocol must also be provided in the manuscript.

Plants

Seed stocks	N/A.
Novel plant genotypes	N/A.
Authentication	N/A.

ChIP-seq

Data deposition

- [x] Confirm that both raw and final processed data have been deposited in a public database such as GEO.
- [ ] Confirm that you have deposited or provided access to graph files (e.g. BED files) for the called peaks.

Data access links
May remain private before publication.

GEO Superseries: GSE279066

Files in database submission

GEO_Superseries GEO_Subseries GEO_Sample Processed_Data_File
GSE279066 GSE262377 GSM8164147 Parent_input.bw
GSE279066 GSE262377 GSM8164149 M3_input.bw
GSE279066 GSE262377 GSM8164151 C10_input.bw
GSE279066 GSE262377 GSM8164153 C10_M3_input.bw
GSE279066 GSE262377 GSM8164155 n2_Parent_Input.bw
GSE279066 GSE262377 GSM8164157 n2_M3_Input.bw
GSE279066 GSE262377 GSM8164159 n2_C10_Input.bw
GSE279066 GSE262377 GSM8164161 n2_C10_M3_Input.bw
GSE279066 GSE262377 GSM8164148 Parent_H3K4me3.bw
GSE279066 GSE262377 GSM8164150 M3_H3K4me3.bw
GSE279066 GSE262377 GSM8164152 C10_H3K4me3.bw
GSE279066 GSE262377 GSM8164154 C10_M3_H3K4me3.bw
GSE279066 GSE262377 GSM8164156 n2_Parent_H3K4me3.bw
GSE279066 GSE262377 GSM8164158 n2_M3_H3K4me3.bw
GSE279066 GSE262377 GSM8164160 n2_C10_H3K4me3.bw
GSE279066 GSE262377 GSM8164162 n2_C10_M3_H3K4me3.bw
GSE279066 GSE279413 GSM8569545 RKI1_NTC_UT_input_n1.bw
GSE279066 GSE279413 GSM8569546 RKI1_NTC_CMPD1_input_n1.bw
GSE279066 GSE279413 GSM8569547 RKI1_PRDM9_KO_UT_input_n1.bw
GSE279066 GSE279413 GSM8569548 RKI1_PRDM9_KO_CMPD1_input_n1.bw
GSE279066 GSE279413 GSM8569549 RKI1_NTC_UT_input_n2.bw
GSE279066 GSE279413 GSM8569550 RKI1_NTC_CMPD1_n2_input_n2.bw
GSE279066 GSE279413 GSM8569551 RKI1_PRDM9_KO_UT_n2_input_n2.bw
GSE279066 GSE279413 GSM8569552 RKI1_PRDM9_KO_CMPD1_n2_input_n2.bw
GSE279066 GSE279413 GSM8569553 RKI1_NTC_UT_H3K4me3_n1.bw
GSE279066 GSE279413 GSM8569554 RKI1_NTC_CMPD1_H3K4me3_n1.bw
GSE279066 GSE279413 GSM8569555 RKI1_PRDM9_KO_UT_H3K4me3_n1.bw
GSE279066 GSE279413 GSM8569556 RKI1_PRDM9_KO_CMPD1_H3K4me3_n1.bw
GSE279066 GSE279413 GSM8569557 RKI1_NTC_UT_H3K4me3_n2.bw
GSE279066 GSE279413 GSM8569558 RKI1_NTC_CMPD1_H3K4me3_n2.bw
GSE279066 GSE279413 GSM8569559 RKI1_PRDM9KO_UT_H3K4me3_n2.bw
GSE279066 GSE279413 GSM8569560 RKI1_PRDM9KO_CMPD1_H3K4me3_n2.bw

Genome browser session
(e.g. UCSC)

hg38 (GSE262377 + GSE279413):
https://genome.ucsc.edu/s/George_Joun/NCOMMS%2D24%2D67473_hg38

hg19 (GSE121723):
https://genome.ucsc.edu/s/George_Joun/NCOMMS%2D24%2D67473_hg19

Methodology

Replicates

n=2 biological replicates, per experiment.

Sequencing depth

GEO_Superseries GEO_Subseries GEO_Sample SampleID Total_Reads Uniquely_Mapped_Reads Length_of_Reads Paired_End/Single_End
GSE279066 GSE262377 GSM8164147 RKI1_n1_Parent_Input 36863538 10866601 50 Single End
GSE279066 GSE262377 GSM8164149 RKI1_n1_MRK-740_Input 74895048 26978359 50 Single End
GSE279066 GSE262377 GSM8164151 RKI1_n1_CMPD1_Input 51974309 37039103 50 Single End
GSE279066 GSE262377 GSM8164153 RKI1_n1_CMPD1+MRK-740_Input 50692332 18764329 50 Single End
GSE279066 GSE262377 GSM8164155 RKI1_n2_Parent_Input 64202260 19578002 50 Single End
GSE279066 GSE262377 GSM8164157 RKI1_n2_MRK-740_Input 73186283 33353099 50 Single End
GSE279066 GSE262377 GSM8164159 RKI1_n2_CMPD1_Input 55844324 16434503 50 Single End
GSE279066 GSE262377 GSM8164161 RKI1_n2_CMPD1+MRK-740_Input 69244022 23817981 50 Single End
GSE279066 GSE262377 GSM8164148 RKI1_n1_Parent_H3K4me3 55454070 42889240 50 Single End
GSE279066 GSE262377 GSM8164150 RKI1_n1_MRK-740_H3K4me3 33276413 25480295 50 Single End
GSE279066 GSE262377 GSM8164152 RKI1_n1_CMPD1_H3K4me3 50402830 39359651 50 Single End
GSE279066 GSE262377 GSM8164154 RKI1_n1_CMPD1+MRK-740_H3K4me3 38247875 28678841 50 Single End

GSE279066 GSE262377 GSM8164156 RKI1_n2_Parent_H3K4me3 43944693 34199544 50 Single End
GSE279066 GSE262377 GSM8164158 RKI1_n2_MRK-740_H3K4me3 37881954 28103234 50 Single End
GSE279066 GSE262377 GSM8164160 RKI1_n2_CMPD1_H3K4me3 32266748 25466618 50 Single End
GSE279066 GSE262377 GSM8164162 RKI1_n2_CMPD1+MRK-740_H3K4me3 29988142 23233017 50 Single End
GSE279066 GSE279413 GSM8569545 RKI1_NTC UT n=1 INPUT 51088171 36841577 50 Single End
GSE279066 GSE279413 GSM8569546 RKI1_NTC C10 n=1 INPUT 56926247 41172996 50 Single End
GSE279066 GSE279413 GSM8569547 RKI1_PRDM9 (1) UT n=1 INPUT 49968957 36061979 50 Single End
GSE279066 GSE279413 GSM8569548 RKI1_PRDM9 (1) C10 n=1 INPUT 43436214 31393640 50 Single End
GSE279066 GSE279413 GSM8569549 RKI1_NTC UT n=2 INPUT 49976990 36087911 50 Single End
GSE279066 GSE279413 GSM8569550 RKI1_NTC C10 n=2 INPUT 60665498 43809456 50 Single End
GSE279066 GSE279413 GSM8569551 RKI1_PRDM9 (1) UT n=2 INPUT 42060997 30277482 50 Single End
GSE279066 GSE279413 GSM8569552 RKI1_PRDM9 (1) C10 n=2 INPUT 66367209 47903918 50 Single End
GSE279066 GSE279413 GSM8569553 RKI1_NTC UT n=1 H3K4me3 ChIP 75596294 55020248 50 Single End
GSE279066 GSE279413 GSM8569554 RKI1_NTC C10 n=1 H3K4me3 ChIP 53054108 38148984 50 Single End
GSE279066 GSE279413 GSM8569555 RKI1_PRDM9 (1) UT n=1 H3K4me3 ChIP 65475483 47692582 50 Single End
GSE279066 GSE279413 GSM8569556 RKI1_PRDM9 (1) C10 n=1 H3K4me3 ChIP 46084138 33001575 50 Single End
GSE279066 GSE279413 GSM8569557 RKI1_NTC UT n=2 H3K4me3 ChIP 62095403 45708156 50 Single End
GSE279066 GSE279413 GSM8569558 RKI1_NTC C10 n=2 H3K4me3 ChIP 45564330 32706671 50 Single End
GSE279066 GSE279413 GSM8569559 RKI1_PRDM9 (1) UT n=2 H3K4me3 ChIP 45167753 32813785 50 Single End
GSE279066 GSE279413 GSM8569560 RKI1_PRDM9 (1) C10 n=2 H3K4me3 ChIP 63485173 45631790 50 Single End

Antibodies

For H3K4me3 ChIPseq:

Cell Signaling Technology: H3K4me3 (Cat# 9751S, Rabbit Monoclonal Antibody): https://www.antibodyregistry.org/AB_2616028

Peak calling parameters

Read mapping was carried out using Bowtie2 on Galaxy (usegalaxy.org.au). Reads were aligned to human genome version hg38.

Peak calling was carried out with MACS2.1.2 callpeak on Galaxy (usegalaxy.org.au). Briefly, alignment results for ChIP and Input were provided, peak calling was carried out with effective human genome size 2.7e9, bandwidth 300, and q-value cutoff of 0.01.

Data quality

To ensure data quality, sequencing run reports were downloaded, and checked, examining parameters such as Q30, index mismatch rates, reads mapping to each sample. Furhermore, FastQC reports were generated across random samples in both experiments to ensure data quality.

Regarding MACS2.1.2 peak calling, a q-value of 0.01 was designated as the cutoff. Peak enrichments were accepted between 10-fold and 30-fold. From this criterion, we received the following number of unique peaks (n_Peaks).

GEO_Superseries GEO_Subseries GEO_Sample SampleID_Comparison n_Peaks
GSE279066 GSE262377 GSM8164148_GSM8164147 RKI1_n1_Parent_H3K4me3 vs RKI1_n1_Parent_Input 27431
GSE279066 GSE262377 GSM8164150_GSM8164149 RKI1_n1_MRK-740_H3K4me3 vs RKI1_n1_MRK-740_Input 26816
GSE279066 GSE262377 GSM8164152_GSM8164151 RKI1_n1_CMPD1_H3K4me3 vs RKI1_n1_CMPD1_Input 27839
GSE279066 GSE262377 GSM8164154_GSM8164153 RKI1_n1_CMPD1+MRK-740_H3K4me3 vs RKI1_n1_CMPD1+MRK-740_Input 28303
GSE279066 GSE262377 GSM8164156_GSM8164155 RKI1_n2_Parent_H3K4me3 vs RKI1_n2_Parent_Input 30772
GSE279066 GSE262377 GSM8164158_GSM8164157 RKI1_n2_MRK-740_H3K4me3 vs RKI1_n2_MRK-740_Input 24289
GSE279066 GSE262377 GSM8164160_GSM8164159 RKI1_n2_CMPD1_H3K4me3 vs RKI1_n2_CMPD1_Input 29962
GSE279066 GSE262377 GSM8164162_GSM8164161 RKI1_n2_CMPD1+MRK-740_H3K4me3 vs RKI1_n2_CMPD1+MRK-740_Input 27045
GSE279066 GSE279413 GSM8569553_GSM8569545 RKI1_NTC UT n=1 H3K4me3 ChIP vs RKI1_NTC UT n=1 INPUT 23073
GSE279066 GSE279413 GSM8569554_GSM8569546 RKI1_NTC C10 n=1 H3K4me3 ChIP vs RKI1_NTC C10 n=1 INPUT 19152
GSE279066 GSE279413 GSM8569555_GSM8569547 RKI1_PRDM9 (1) UT n=1 H3K4me3 ChIP vs RKI1_PRDM9 (1) UT n=1 INPUT 21474
GSE279066 GSE279413 GSM8569556_GSM8569548 RKI1_PRDM9 (1) C10 n=1 H3K4me3 ChIP vs RKI1_PRDM9 (1) C10 n=1 INPUT 139
GSE279066 GSE279413 GSM8569557_GSM8569549 RKI1_NTC UT n=2 H3K4me3 ChIP vs RKI1_NTC UT n=2 INPUT 14075
GSE279066 GSE279413 GSM8569558_GSM8569550 RKI1_NTC C10 n=2 H3K4me3 ChIP vs RKI1_NTC C10 n=2 INPUT 2277
GSE279066 GSE279413 GSM8569559_GSM8569551 RKI1_PRDM9 (1) UT n=2 H3K4me3 ChIP vs RKI1_PRDM9 (1) UT n=2 INPUT 16865
GSE279066 GSE279413 GSM8569560_GSM8569552 RKI1_PRDM9 (1) C10 n=2 H3K4me3 ChIP vs RKI1_PRDM9 (1) C10 n=2 INPUT 51

Software

Python 3.11 command line Deeptools 2.0 pipeline was used for generating population level H3K4me3 peak intensity graphs, as in Figure 3j, 5d, 5f.
Python 3.11 command line PyGenomeTracks was used for graphing H3K4me3 peak signals at specified, individual genome regions, as in Figure 3k, 5i.
Python 3.11 command line MEME Suite 5.5.7 was used for extracting the most significant genomic regions, in BED format, for Homo Sapiens PRDM9 binding motif frequency matrix, in the hg38 and hg19 human genome assembly.

Flow Cytometry

Plots

Confirm that:

- [ ] The axis labels state the marker and fluorochrome used (e.g. CD4-FITC).
- [ ] The axis scales are clearly visible. Include numbers along axes only for bottom left plot of group (a 'group' is an analysis of identical markers).
- [ ] All plots are contour plots with outliers or pseudocolor plots.
- [x] A numerical value for number of cells or percentage (with statistics) is provided.

Methodology

Sample preparation	Samples were prepared per manufacturer's protocol: CellROXTM Green Reagent (ThermoFisher Scientific, Cat# C10444) and Image-iT Lipid Peroxidation kit (Thermofisher Scientific, Cat# C10445). For propidium iodide DNA content measurements, see Methods.
Instrument	BD LSR Fortessa X-20.
Software	BD FACSDiva software, was used to acquire data. FlowJo v10.8.1 was used to analyse data.
Cell population abundance	The median staining intensity was used, per treatment condition, to assess changes in a particular marker between treatment conditions.
Gating strategy	Staining gates were based on (+) and (-) controls. All samples, within a biological repeat, were subjected to identical gating parameters. Single cells were gated for and only included in analysis.

- [ ] Tick this box to confirm that a figure exemplifying the gating strategy is provided in the Supplementary Information.
